# DDB2 represses ovarian cancer cell dedifferentiation by suppressing ALDH1A1

**DOI:** 10.1038/s41419-018-0585-y

**Published:** 2018-05-11

**Authors:** Tiantian Cui, Amit Kumar Srivastava, Chunhua Han, Dayong Wu, Nissar Wani, Lu Liu, Zhiqin Gao, Meihua Qu, Ning Zou, Xiaoli Zhang, Ping Yi, Jianhua Yu, Erica H. Bell, Shyh-Ming Yang, David J. Maloney, Yanfang Zheng, Altaf A. Wani, Qi-En Wang

**Affiliations:** 1 Department of Radiology, College of Medicine, The Ohio State University, Columbus, OH 43210 USA; 2 Department of Pathology, College of Medicine, The Ohio State University, Columbus, OH 43210 USA; 3 Oncology Center, Zhujiang Hospital, Southern Medical University, 510282 Guangdong Guangzhou, China; 4 Department of Cell Biology, Weifang Medical University, 264053 Shandong Weifang, China; 5 Department of Pharmacology, Weifang Medical University, 264053 Shandong Weifang, China; 6 Department of Radiation Oncology, Hubei Cancer Hospital, 430079 Hubei Wuhan, China; 7 Center for Biostatistics, College of Medicine, The Ohio State University, Columbus, OH 43210 USA; 8 Department of Obstetrics and Gynecology, Daping Hospital, The Third Military Medical University, 40042 Chongqing, China; 9 Department of Internal Medicine, Division of Hematology, College of Medicine, The Ohio State University, Columbus, OH 43210 USA; 10 Department of Radiation Oncology, College of Medicine, The Ohio State University, Columbus, OH 43210 USA; 11 National Center for Advancing Translational Science, National Institutes of Health, Rockville, MD 20850 USA; 12 Present Address: Department of Biotechnology, CSIR-North East Institute of Science and Technology (CSIR-NEIST), Jorhat, Assam 785006 India

## Abstract

Cancer stem cells (CSCs), representing the root of many solid tumors including ovarian cancer, have been implicated in disease recurrence, metastasis, and therapeutic resistance. Our previous study has demonstrated that the CSC subpopulation in ovarian cancer can be limited by DNA damage-binding protein 2 (DDB2). Here, we demonstrated that the ovarian CSC subpopulation can be maintained via cancer cell dedifferentiation, and DDB2 is able to suppress this non-CSC-to-CSC conversion by repression of *ALDH1A1* transcription. Mechanistically, DDB2 binds to the *ALDH1A1* gene promoter, facilitating the enrichment of histone H3K27me3, and competing with the transcription factor C/EBPβ for binding to this region, eventually inhibiting the promoter activity of the *ALDH1A1* gene. The de-repression of ALDH1A1 expression contributes to DDB2 silencing-augmented non-CSC-to-CSC conversion and expansion of the CSC subpopulation. We further showed that treatment with a selective ALDH1A1 inhibitor blocked DDB2 silencing-induced expansion of CSCs, and halted orthotopic xenograft tumor growth. Together, our data demonstrate that DDB2, functioning as a transcription repressor, can abrogate ovarian CSC properties by downregulating ALDH1A1 expression.

## Introduction

Ovarian cancer is the most lethal malignancy of the female reproductive tract with a poor 5-year survival rate of only 28% in advanced stages, at which, 60% of cases are diagnosed^1^. Most tumors are initially responsive to conventional chemotherapy, and go into clinical remission after initial treatment. However, tumor metastasis and recurrence occur in >70% of ovarian cancer patients despite treatment, ultimately leading to death^2^. Therefore, identifying efficient ways to halt ovarian cancer progression is particularly important to improving progression-free survival and decreasing the mortality in ovarian cancer patients.

Over the past few years, growing evidence suggests that the presence of cancer stem cells (CSCs) is the most important trigger of tumor initiation and progression^3–5^. These CSCs, with enhanced tumorigenicity and chemoresistance, have been identified in a variety of solid tumors including ovarian cancer^6–9^, and are considered to be responsible for treatment failure, tumor metastasis, and recurrence. Thus, eradication of CSCs could be an effective way to improve therapeutic efficacy.

DNA damage-binding protein 2 (DDB2) has been considered a tumor suppressor based on the findings that DDB2-knockout mice were not only susceptible to UV-induced skin cancer, but also more vulnerable to spontaneous malignant neoplasms^10,11^. DDB2 is also able to enhance cellular apoptosis through downregulation of Bcl-2^12,13^ and p21^14^; inhibit colon tumor metastasis through blockage of epithelial-mesenchymal transition (EMT)^15^; limit the motility and invasiveness of invasive human breast tumor cells by regulating NF-κB activity^16^, as well as mediate premature senescence^17^. Low *DDB2* mRNA expression in ovarian tumors correlates with poor outcome of ovarian cancer patients^18^, and similar findings were also found in breast cancer patients^16^. In addition, DDB2 has been demonstrated to suppress the tumorigenicity of ovarian cancer cells^18^ and colorectal cancer cells^15^. Our previous study has shown that DDB2 can reduce the abundance of CSCs, which are characterized by enhanced activity of high aldehyde dehydrogenase activity (ALDH^+^) or CD44^+^CD117^+^, in ovarian cancer cell lines, providing a novel mechanism to explain the DDB2-mediated suppression of tumorigenicity, and also suggesting that low expression of DDB2 is essential to maintenance of CSC properties^18^.

High ALDH activity is observed in CSCs of multiple cancer types, and is often used to isolate and functionally characterize CSCs^18–21^. ALDH1A1 is a member of the highly conserved ALDH family, which includes 19 enzymes involved in the metabolism of chemicals that are critical to stem cell self-renewal and/or differentiation^22^. ALDH1A1 also plays a critical role in the regulation of the CSC subpopulation^23,24^. The expression and activity of ALDH1A1 can be regulated by β-Catenin^23^, the NOTCH pathway^25^, enhancer of zeste 2 polycomb repressive complex 2 (EZH2)^26^, and the bromodomain and extraterminal (BET) family of proteins^27^. Interestingly, our previous microarray analysis suggests that ALDH1A1 could be a target gene downregulated by DDB2^28^. However, this relationship has yet to be validated and the underlying mechanism remains unclear.

Similar to normal stem cells, CSCs also possess capacity to self-renew and differentiate into heterogeneous cancer cells. However, CSCs may not necessarily originate from normal tissue stem cells or progenitor cells^29^. It has been recently reported that normal and neoplastic epithelial cells can re-enter the stem cell state^30^. This tumor cell plasticity enables non-CSCs to dedifferentiate and acquire CSC-like properties under certain conditions. Here, we demonstrate that cancer cell dedifferentiation indeed occurs in ovarian cancer cell lines. DDB2 can inhibit the ovarian cancer cell dedifferentiation through downregulation of ALDH1A1; a selective ALDH1A1 inhibitor is able to reduce the tumorigenic CSC subpopulation and halt tumor growth in ovarian cancer cells possessing low levels of DDB2.

## Results

### DDB2 inhibits non-CSC-to-CSC conversions in ovarian cancer

**Fig. 1:**
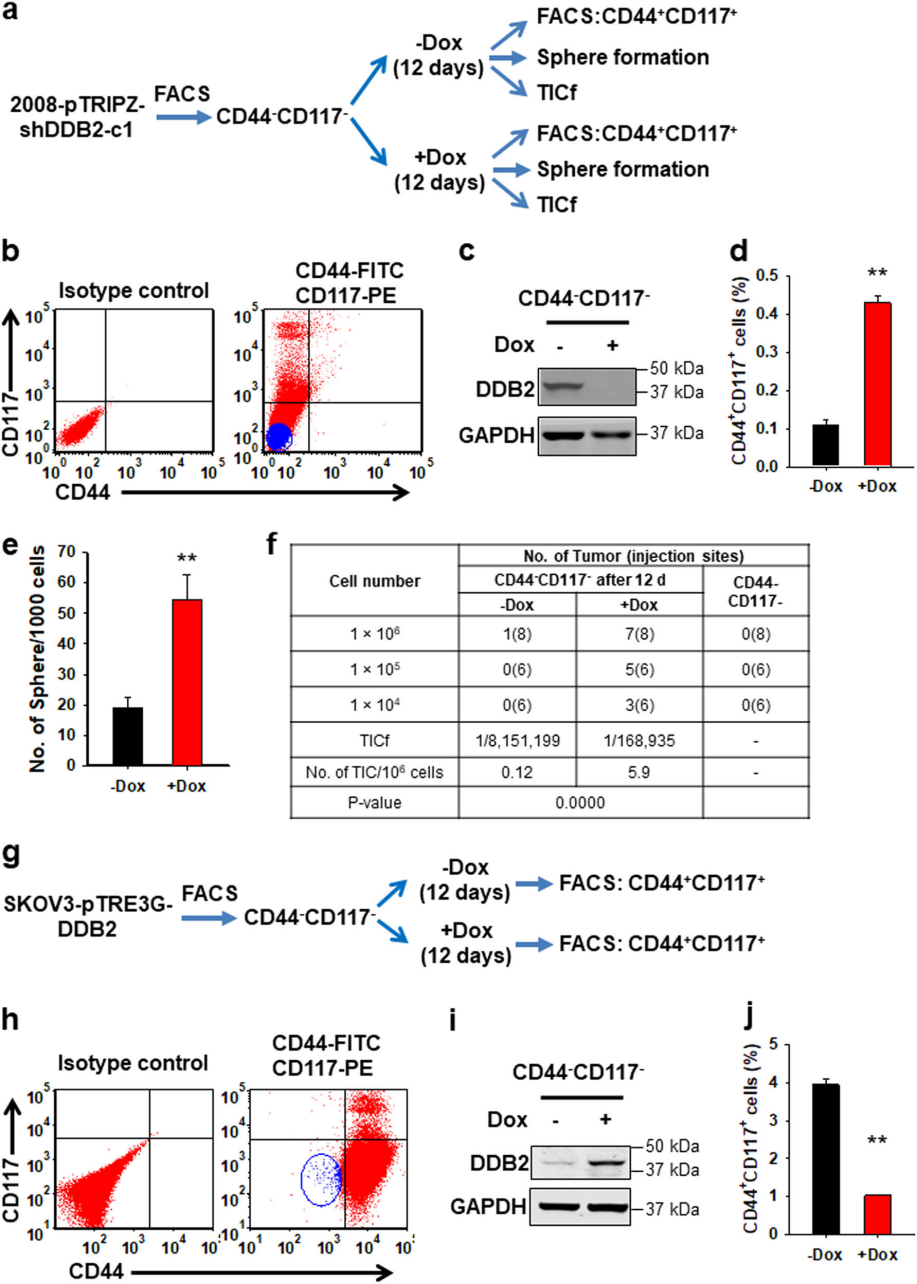
DDB2 negatively regulates the conversion of non-CSCs to CSCs in ovarian cancer cells. **a** Schematic outline of the experimental procedure in Tet-inducible DDB2 knockdown cells (2008-pTRIPZ-shDDB2). **b** Sorting of CD44^−^CD117^−^ cells (blue) from 2008-pTRIPZ-shDDB2-c1 cells. **c**–**f** CD44^−^CD117^−^ cells were cultured in the absence or presence of Dox for 12 days, DDB2 expression was determined using immunoblotting (**c**), the percentage of CD44^+^CD117^+^ cells was determined using FACS (**d**), the sphere formation ability was determined using the sphere-forming assay (**e**). *N* = 3, Bar: SD, **P < 0.01. The frequency of tumor-initiating cells (TICf) was quantified by a xenograft assay with limiting dilution, and calculated using the ELDA software (**f**). **g** Schematic outline of the experimental procedure in Tet-inducible DDB2 overexpression cells (SKOV3-pTRE3G-DDB2). **h** Sorting of CD44^−^CD117^−^ cells (blue) from SKOV3-pTRE3G-DDB2 cells. **i**, **j** CD44^−^CD117^−^ cells were cultured in the presence or absence of Dox for 12 days, DDB2 expression was determined using immunoblotting (**i**), the percentage of CD44^+^CD117^+^ cells was determined using FACS (**j**). *N* = 3, Bar: SD, **P < 0.01

Given that the CSC subpopulation in a tumor can be maintained by non-CSC dedifferentiation^30,31^, we attempted to determine whether non-CSC dedifferentiation exists in ovarian cancer cells, and whether DDB2 silencing expands the CSC subpopulation by promoting non-CSC-to-CSC conversions. We transfected Tet-On pTRIPZ-inducible shDDB2 plasmids into an ovarian cancer cell line 2008, established two Tet-On-inducible *DDB2* downregulation cell lines, 2008-pTRIPZ-shDDB2, and confirmed the effect of DDB2 silencing on the expansion of CSC population (Supplementary Figure S1a–h). We further purified CD44^−^CD117^−^ cells from 2008-pTRIPZ-shDDB2-c1 cells using fluorescence-activated cell sorting (FACS) (Fig. 1a, b), cultured them in the absence or presence of Doxycycline (Dox) to modulate DDB2 expression level for 12 days, and determined the emergence of CD44^+^CD117^+^ cells, which have been characterized to possess CSC properties^18^. We indeed found that CD44^−^CD117^−^ cells can convert to CD44^+^CD117^+^ cells, and this conversion can be promoted by Dox-induced DDB2 downregulation (Fig. 1c, d).

To functionally ascertain the effect of DDB2 silencing on the non-CSC-to-CSC conversion, we analyzed the sphere formation rate and the frequency of tumor-initiating cells (TICf) in the CD44^−^CD117^−^ cells after 12 days of culture in the absence or presence of Dox. DDB2 knockdown (+Dox) enhanced the in vitro sphere formation rate of those CD44^−^CD117^−^ cells after 12 days of culture (Fig. 1e). The CD44^−^CD117^−^ cells, which lack tumorigenicity in immunodeficient mice (Fig. 1f, right panel), gained tumorigenic potential after 12 days of culture (Fig. 1f, left panel), validating the existence of non-CSC-to-CSC conversions. Notably, DDB2 knockdown (+Dox) enhanced the tumorigenic potential of those CD44^−^CD117^−^ cells after 12 days of culture (Fig. 1f, middle panel), further supporting the role of DDB2 silencing in promoting non-CSC-to-CSC conversions in this ovarian cancer cell line. This DDB2-mediated suppression of cancer cell dedifferentiation was also confirmed in another ovarian cancer cell line SKOV3 possessing Dox-inducible DDB2 overexpression (Fig. 1g–j).

**Fig. 2:**
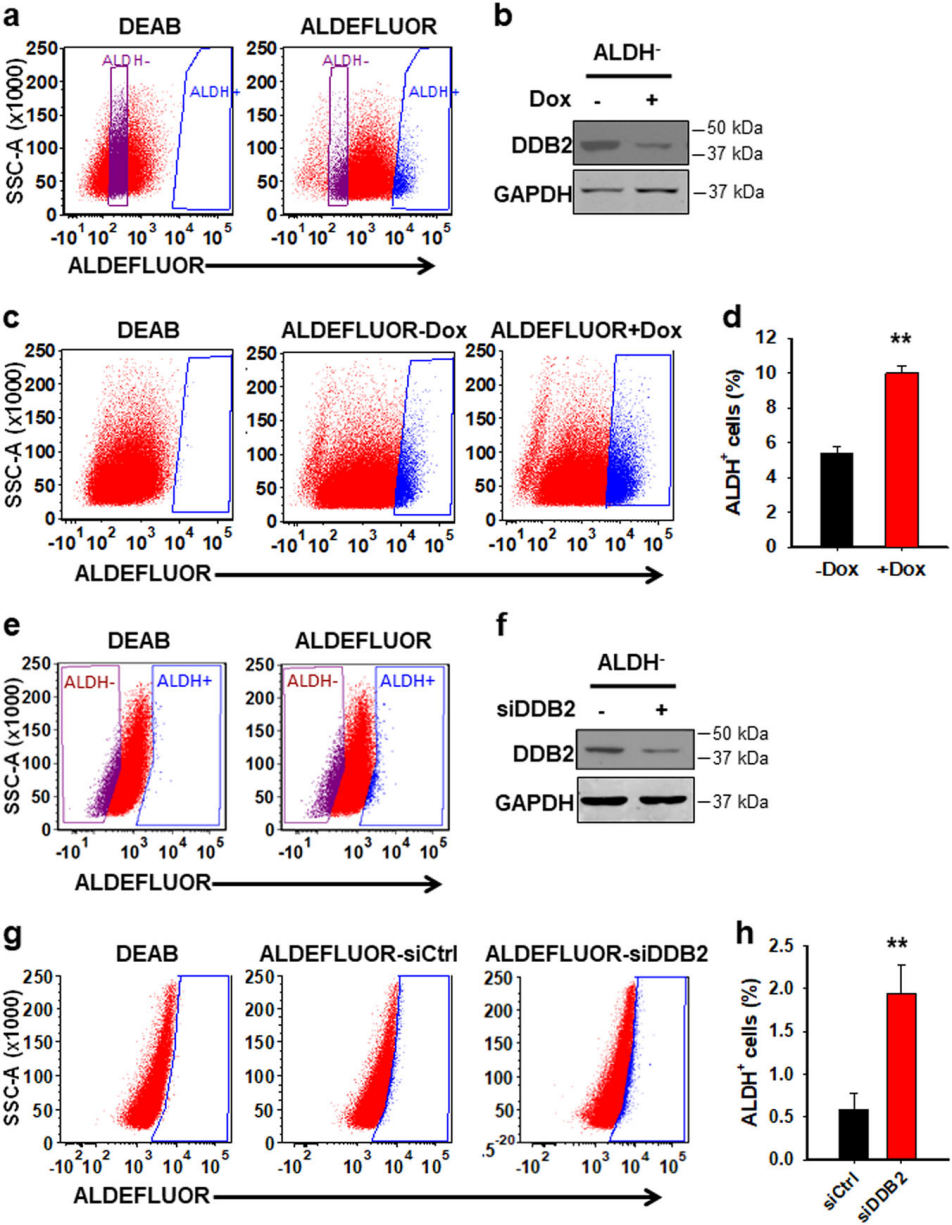
Downregulation of DDB2 promotes the conversion of ALDH^−^ to ALDH^+^ cells in ovarian cancer cells. **a** Sorting of ALDH^−^ cells (purple) from 2008-pTRIPZ-shDDB2-c1 cells. **b**–**d** ALDH^−^ cells were cultured in the absence or presence of Dox for 12 days, DDB2 expression was determined using immunoblotting (**b**), the percentage of ALDH^+^ cells (blue) was determined using FACS (**c**, **d**). *N* = 3, Bar: SD, **P < 0.01. **e** Sorting of ALDH^−^ cells (purple) from OVCAR4 cells. **f**–**h** ALDH^−^ cells were cultured for 12 days, and transfected with control or DDB2 siRNA every 3 days during this period. DDB2 expression was determined using immunoblotting (**f**), the percentage of ALDH^+^ cells (blue) was determined using FACS (**g**, **h**). *N* = 3, Bar: SD, **P < 0.01. Note: DEAB was used as a negative control for gating of ALDH^+^ cells

Besides CD44^+^CD117^+^ cells, we have also demonstrated that ALDH^+^ cells isolated from the ovarian cancer cell line 2008 have all known CSC properties including high tumor-initiating capacity (Supplementary Figure S2), and DDB2 silencing is able to increase the ALDH^+^ cell population in these cells (Supplementary Figure S1b, c, e and f). To determine whether DDB2 can affect the conversions of ALDH^−^ cells to ALDH^+^ cells as well, we isolated ALDH^−^ cells from 2008-pTRIPZ-shDDB2 cells, cultured them in the absence or presence of Dox for 12 days, and determined the emergence of ALDH^+^ cells. Same as aforementioned conversion of CD44^−^CD117^−^ cells to CD44^+^CD117^+^ cells, ALDH^+^ cells can be produced de novo from ALDH^−^ cells, and Dox-induced DDB2 silencing, but not Dox itself, promoted this process (Fig. 2a–c, Supplementary Figure S3). We further confirmed this finding by using one of the most likely HGSOC cell line OVCAR4^32^. As shown in Figure 2e–h, knockdown of DDB2 increased the conversion of ALDH^−^ cells to ALDH^+^ cells in OVCAR4 cells. In addition, we isolated several single cell clones from another ovarian cancer cell line CP70, sorted ALDH^−^ cells from the C6 cell clone possessing low level of DDB2 and the C19 cell clone possessing high level of DDB2, respectively (Supplementary Figure S4a–c), and further cultured them for 12 days. We again found more de novo-produced ALDH^+^ cells in the low DDB2-expressing C6 clone than that in the high DDB2-expressing C19 clones (Supplementary Figure S4b, c, e). Furthermore, knockdown of DDB2 expression in C19-derived ALDH^−^ cells increased the de novo production of ALDH^+^ cells (Supplementary Figure S4c, d, e). Taken together, these data indicate that DDB2 silencing is able to facilitate the ovarian cancer cell dedifferentiation, characterized by both CD44^−^CD117^−^ to CD44^+^CD117^+^ cell conversions and ALDH^−^ to ALDH^+^ cell conversions, as well as the non-tumorigenic to tumorigenic cell conversions.

### DDB2 negatively regulates ALDH1A1 expression

**Fig. 3:**
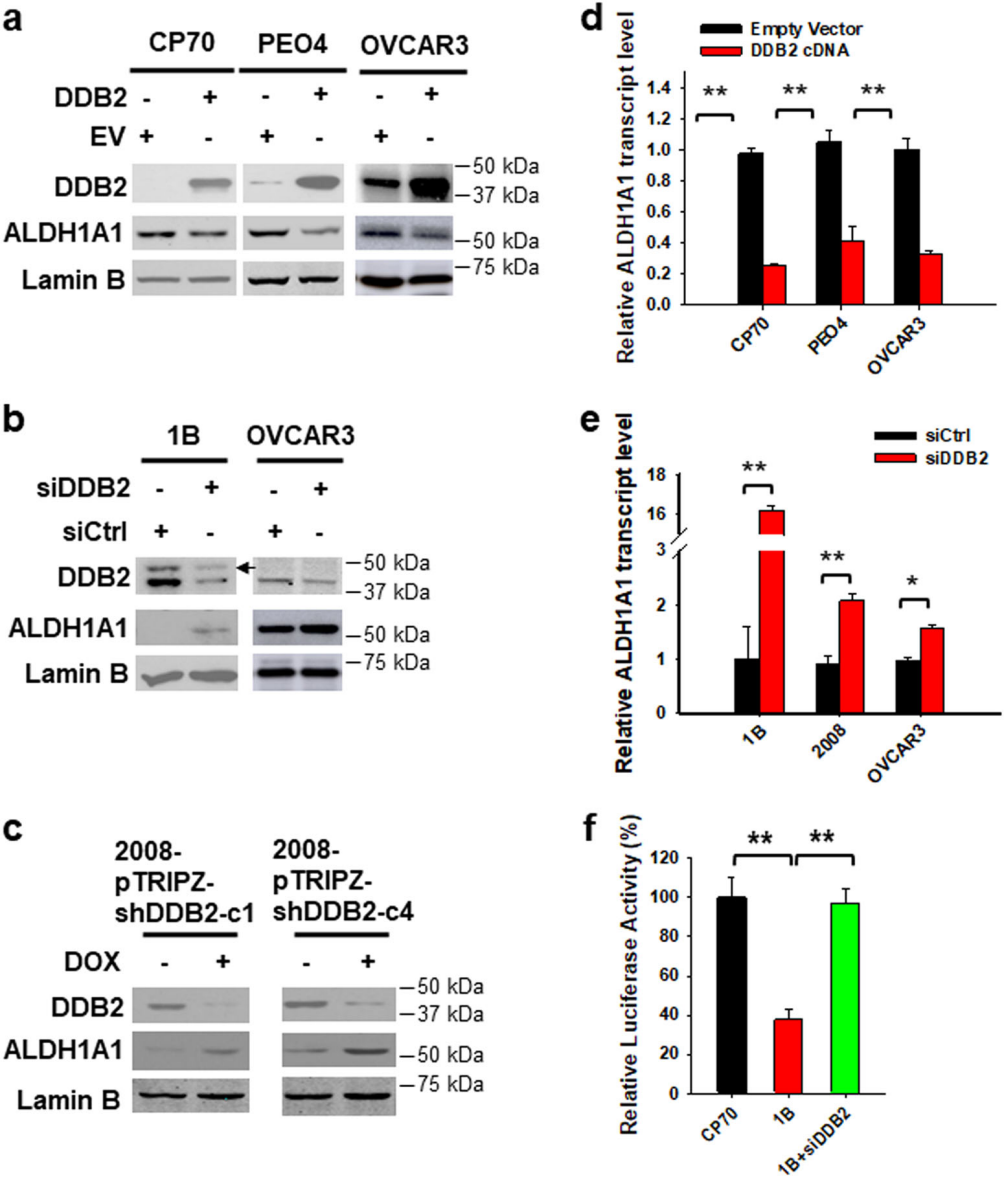
DDB2 downregulates ALDH1A1 expression. **a**–**c** Alteration of ALDH1A1 protein levels in various ovarian cancer cell lines after modulation of DDB2 expression. CP70, PEO4, and OVCAR3 cells were transiently transfected with DDB2-expressing vectors for 48 h (**a**); DDB2 stably expressing CP70-DDB2-1B (1B) cells and OVCAR3 cells were transiently transfected with DDB2 siRNA for 48 h (**b**) (arrow: His-Xpress-tagged DDB2); Tet-inducible 2008-pTRIPZ-shDDB2 cells were treated with Dox for 72 h (**c**). The protein levels of DDB2 and ALDH1A1 were determined using immunoblotting. Lamin B was also determined to serve as a loading control. **d**, **e** Alteration of *ALDH1A1* mRNA levels in various ovarian cancer cell lines after modulation of DDB2 expression. CP70, PEO4, and OVCAR3 cells were transiently transfected with DDB2-expressing vectors for 48 h (**d**); DDB2 stably expressing CP70-DDB2-1B (1B), 2008, and OVCAR3 cells were transiently transfected with DDB2 siRNA for 48 h (**e**). The mRNA level of *ALDH1A1* was determined using qRT-PCR. **f** The promoter activity of the *ALDH1A1* gene was determined using the luciferase reporter assay in CP70 cells, DDB2 stably transfected CP70 (1B) cells, and 1B cells transiently transfected with DDB2 siRNA. *N* = 3, Bar: SD, **P < 0.01

Our previous microarray analysis has suggested that *ALDH1A1*, an isoenzyme of *ALDH1*, is a downstream gene negatively regulated by DDB2 (GSE66636)^28^. We have also found that ALDH1A1 expression reduced in two clones of DDB2 stably overexpressed CP70 cells^18^. Given that ALDH1 is believed to be a major contributor to the enhanced ALDH activity in ovarian cancer^33^, and high ALDH activity plays a critical role in maintenance of the CSC subpopulation^24^, we reasoned that DDB2 silencing may promote ovarian cancer cell dedifferentiation by enhancing ALDH1A1 expression. To ascertain the role of DDB2 in the regulation of ALDH1A1 expression, we overexpressed DDB2 in ovarian cancer cell lines possessing low DDB2 expression, or downregulated DDB2 in ovarian cancer cell lines possessing high DDB2 expression. We indeed found that overexpression of DDB2 reduced the expression of ALDH1A1, while knockdown of DDB2 increased the expression of ALDH1A1, at both protein (Fig. 3a–c) and mRNA levels (Fig. 3d, e). Furthermore, the luciferase reporter assay demonstrated that DDB2 can inhibit the promoter activity of the *ALDH1A1* gene (Fig. 3f). These data confirm that ALDH1A1 expression can be negatively regulated by DDB2 in ovarian cancer cells.

### DDB2 binds to the *ALDH1A1* gene promoter, functioning as a transcription repressor

**Fig. 4:**
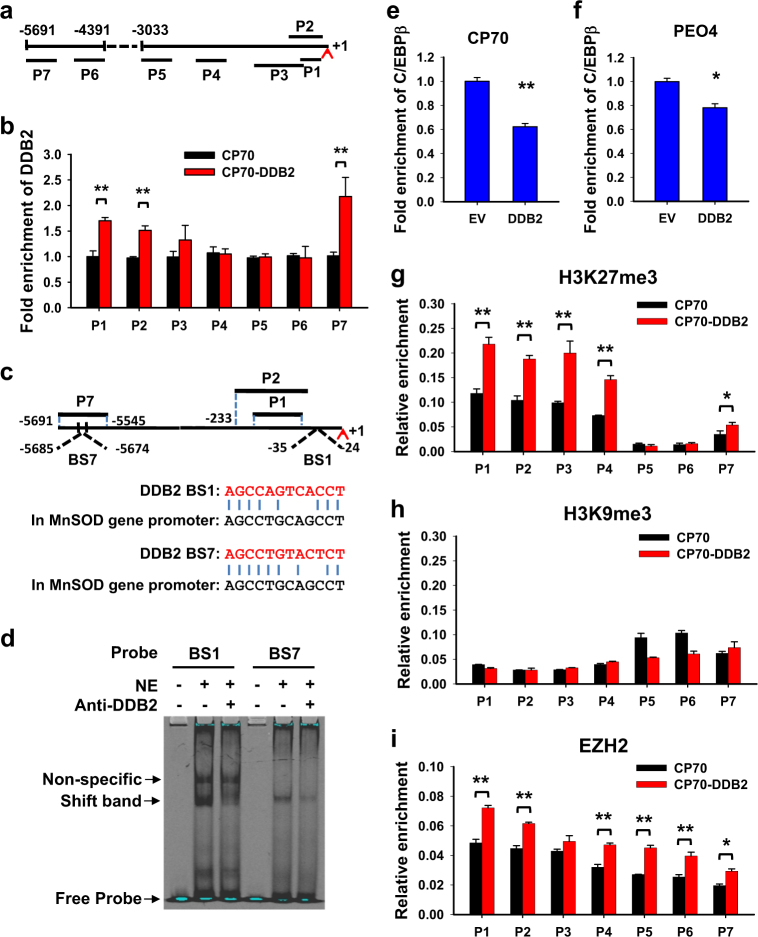
DDB2 binds to the *ALDH1A1* promoter, interfering with C/EBPβ binding and enhancing the enrichment of H3K27me3. **a** Schematic depiction of the *ALDH1A1* promoter region and the regions covered by primer sets. **b** The ChIP assay was conducted in CP70 cells transiently transfected with FLAG-tagged DDB2 to analyze the local enrichment of DDB2 across the *ALDH1A1* promoter region. **c** The schematic representation of the *ALDH1A1* promoter region with putative DDB2 binding sites (BS1 and BS7). **d** The EMSA was performed to determine the direct binding of DDB2 to the *ALDH1A1* promoter. Nuclear extract (NE) from HeLa cells (8 µg) were incubated with IR700-labeld oligo probes. Anti-DDB2 antibody (2 µg) was also included for supershift analysis. **e**, **f** The ChIP analysis with anti-C/EBPβ antibody was conducted in CP70 (**e**) and PEO4 cells (**f**) transiently transfected with empty vector (EV) or DDB2-expressing constructs to analyze the effect of DDB2 on the enrichment of C/EBPβ to the *ALDH1A1* promoter region. **g**–**i** The ChIP assay was carried out in CP70 cells transiently transfected with empty vector or DDB2-expressing constructs to analyze the local enrichment of H3K27me3 (**g**), H3K9me3 (**h**), and EZH2 (**i**) across the ALDH1A1 promoter region. *N* = 3, Bar: SD, *P < 0.05, **P < 0.01

DDB2 has been recognized as a transcription regulator and is able to bind to the promoter region of its target genes^13,15–17,28,34^. Using the chromatin immunoprecipitation (ChIP) assay, we found that DDB2 can also bind to the promoter region of the *ALDH1A1* gene (Fig. 4a, b). It has been reported that DDB2 can repress MnSOD2 transcription via a cis-response element 5′-AGCCTGCAGCCT-3′ located in the proximal promoter of the *MnSOD2* gene^34^. Thus, we performed a thorough sequence alignment with this DDB2-binding sequence across the regions around P1, P2, and P7. Two putative DDB2-binding site (BS1 and BS7) were identified (Fig. 4c). To further confirm whether DDB2 binds to these regions directly, the electrophoretic mobility shift assay (EMSA) was performed using two oligos corresponding to these putative DDB2-binding regions. As shown in Figure 4d, addition of nuclear extracts caused a slower-migrating species in both probes. The intensity of these slower-migrating species reduced after anti-DDB2 antibody was added to the system, indicating that DDB2 can directly bind to these putative DDB2-binding sites.

The *ALDH1A1* gene promoter includes a CCAAT box in the proximal region upstream to the transcription start site^35,36^, and can be transactivated by the binding of C/EBPβ transcription activator^36^. Given that DDB2 is able to bind to the proximal promoter of the *ALDH1A1* gene, we sought to determine whether DDB2 interferes with C/EBPβ binding in this region. By performing the ChIP assay with anti-C/EBPβ antibody in CP70 and PEO4 cells transiently transfected with DDB2, we found that overexpression of DDB2 reduced the enrichment of C/EBPβ to this region (Fig. 4e, f), indicating that DDB2 could compete with the transcription factor C/EBPβ for binding to the promoter region of the *ALDH1A1* gene.

Given that DDB2 can bind to the promoter to regulate the local histone modifications, especially histone H3 trimethylation status, around the promoter region to affect the promoter activity of these genes^13,15,17,28^, we determined the effect of DDB2 on histone H3 trimethylation status in the promoter region of the *ALDH1A1* gene. The ChIP analyses revealed that overexpression of DDB2 increased the local enrichment of histone H3 trimethylation at lysine 27 (H3K27me3), but not trimethylated histone H3 at lysine 9 (H3K9me3), to the entire promoter region (Fig. 4g, h). It is known that EZH2 is a histone methyltransferase that specifically catalyze histone H3K27 trimethylation to mediate gene silencing including *ALDH1A1*^26,37,38^. In addition, we have demonstrated that DDB2 is capable of interacting with EZH2 and recruiting PRC2 (polycomb repressive complex 2) to the promoter region of the *NEDD4L* gene for catalyzing trimethylation of the local histone H3 at K27^28^. Therefore, we determined whether DDB2 affects the enrichment of EZH2 on the *ALDH1A1* promoter. The ChIP analysis demonstrated that overexpression of DDB2 indeed increased the recruitment of EZH2 to the *ALDH1A1* promoter (Fig. 4i). Taken together, these data indicate that DDB2 recruits EZH2 to the *ALDH1A1* promoter region, facilitating the trimethylation of local histone H3 at K27, and represses transcription of the *ALDH1A1* gene.

### ALDH1A1 plays a critical role in DDB2 silencing-promoted expansion of ovarian CSCs

**Fig. 5:**
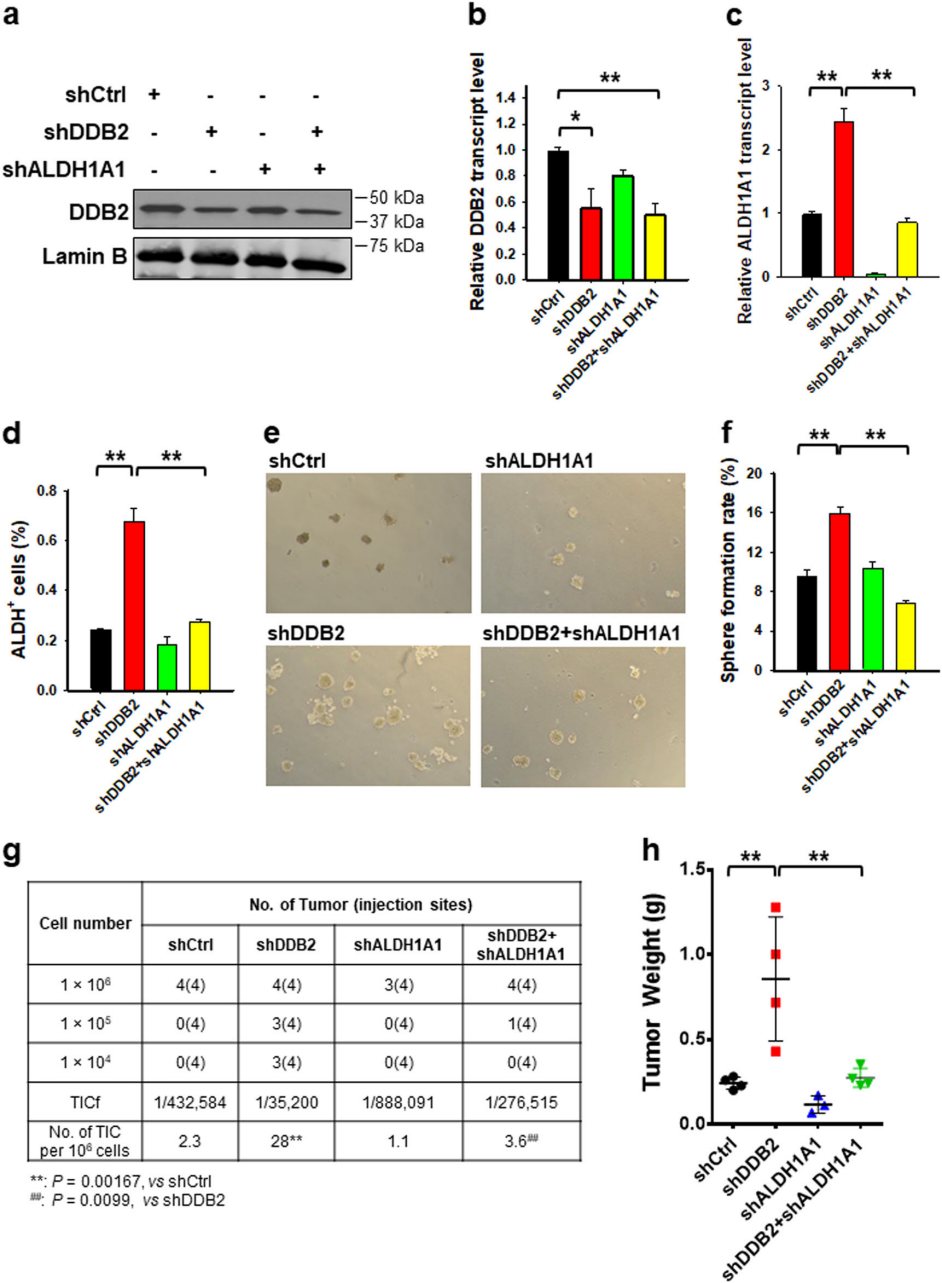
DDB2 silencing expands the ovarian CSC subpopulation via upregulating ALDH1A1 expression. **a**, **b** DDB2 expression at protein (**a**) and mRNA (**b**) levels in 2008 cells after transient transfection with DDB2 or/and ALDH1A1 shRNA for 2 days. *N* = 3, Bar: SD, **P < 0.01. **c** ALDH1A1 mRNA level in 2008 cells after transient transfection with DDB2 or/and ALDH1A1 shRNA for 2 days. *N* = 3, Bar: SD, **P < 0.01. **d** ALDEFLUOR assay was used to determine the percentage of ALDH^+^ cells in 2008 cells after transfection with DDB2 or/and ALDH1A1 shRNA for 2 days. *N* = 3, Bar: SD, **P < 0.01. **e**, **f** Sphere formation ability was determined in 2008 cells after transfection with DDB2 or/and ALDH1A1 shRNA. *N* = 3, Bar: SD, **P < 0.01. **g** The TICf in 2008 cells after transfection with DDB2 or/and ALDH1A1 shRNA was quantified by a xenograft assay with limiting dilution. **h** Tumor weights of xenografts generated with 1 × 10^6^ cells at the end of the xenograft experiment. **P < 0.01

Given that DDB2 silencing is able to enhance ALDH1A1 expression by derepressing its transcription, we attempted to determine whether ALDH1A1 plays an important role in DDB2 silencing-induced expansion of ovarian CSCs. To this end, we knocked down the expression of DDB2 and ALDH1A1 either separately or simultaneously in the 2008 ovarian cancer cell line, and analyzed the change of CSC abundance phenotypically and functionally. Immunoblotting analysis and quantitative reverse-transcription PCR (qRT-PCR) confirmed the downregulation of DDB2 by shDDB2 transfection (Fig. 5a, b). However, due to the extremely low abundance of ALDH^+^ cells in the 2008 ovarian cancer cell line, we were unable to detect the ALDH1A1 protein level using immunoblotting. Instead, we used qRT-PCR to confirm the upregulation of ALDH1A1 by shDDB2 transfection, and downregulation of ALDH1A1 by shALDH1A1 transfection (Fig. 5c). We also used the ALDEFLUOR assay to assess the functional depletion of ALDH1A1 by shALDH1A1 transfection (Fig. 5b). Similar to the previous findings, DDB2 knockdown expanded the ALDH^+^ cell subpopulation; concurrent transfection with shALDH1A1 blocked this ALDH^+^ cell expansion, indicating a successful knockdown of ALDH1A1 in these cells (Fig. 5d). DDB2 knockdown increased the in vitro sphere formation capability and in vivo tumorigenicity of 2008 ovarian cancer cells (Fig. 5e–h), indicating an expansion of CSCs in DDB2 downregulated 2008 cells, which was also shown in Supplementary Figure 1. However, simultaneous knockdown of DDB2 and ALDH1A1 inhibited DDB2 downregulation-enhanced CSC expansion (Fig. 5e–h), indicating that ALDH1A1 mediates DDB2 silencing-promoted expansion of ovarian CSCs.

### ALDH1A1 is essential to DDB2 silencing-augmented cancer cell dedifferentiation

**Fig. 6:**
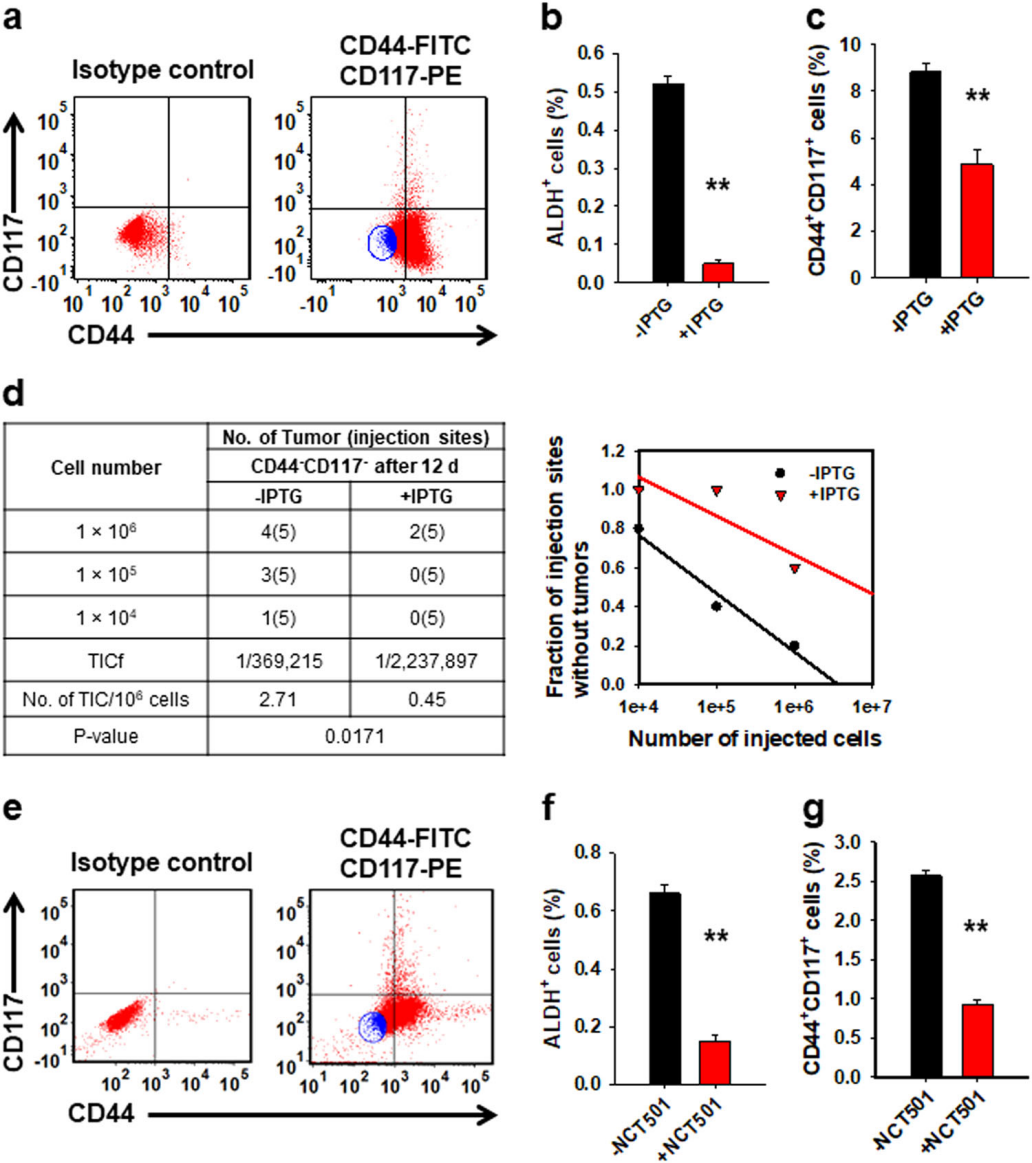
ALDH1A1 is critical to cancer cell dedifferentiation. **a** Sorting of CD44^−^CD117^−^ cells (blue) from 2008-IPTG-shALDH1A1 cells using FACS. **b**–**d** CD44^−^CD117^−^ cells were cultured in the absence or presence of IPTG for 12 days, the percentages of ALDH^+^ cells (**b**) and CD44^+^CD117^+^ cells (**c**) were determined using FACS. *N* = 3, Bar: SD, **P < 0.01. The TICf was quantified by a xenograft assay with limiting dilution (**d**). **e** Sorting of CD44^−^CD117^−^ cells (blue) from 2008 cells using FACS. **f**, **g** CD44^−^CD117^−^ cells were cultured in the absence or presence of the ALDH1A1 inhibitor NCT-501 for 12 days, the percentages of ALDH^+^ cells (**f**) and CD44^+^CD117^+^ cells (**g**) were determined using FACS. *N* = 3, Bar: SD, **P < 0.01

Given that DDB2 silencing promotes the cancer cell dedifferentiation, and ALDH1A1 plays a critical role in DDB2 silencing-induced expansion of the CSC subpopulation, we sought to determine whether ALDH1A1 is essential for DDB2 silencing-augmented non-CSC-to-CSC conversions. We first sorted CD44^−^CD117^−^ cells from a stably transfected ovarian cancer cell line 2008 possessing Isopropyl β-d-1-thiogalactopyranoside (IPTG)-inducible shALDH1A1 (Fig. 6a), and cultured them in the absence or presence of IPTG. Following culture for 12 days, we found that the abundance of ALDH^+^ cells reduced in IPTG-treated cells, confirming the IPTG-induced downregulation of ALDH1A1 (Fig. 6b). Meanwhile, the de novo production of CD44^+^CD117^+^ cells was also compromised by the IPTG treatment (Fig. 6c). More importantly, IPTG treatment also inhibited the de novo production of tumorigenic cells, reflected by a reduced TICf in IPTG-treated CD44^−^CD117^−^ cells after 12 days of culture (Fig. 6d).

We further determined the effect of ALDH1A1 activity inhibition on the non-CSC-to-CSC conversion in the 2008 cancer cell line by treating cells with a potent and selective ALDH1A1 inhibitor NCT-501^39^. We have shown that 100 µM of NCT-501 is able to maximally inhibit ALDH activity without significant cellular toxicity (Supplementary Figure S5). Thus, CD44^−^CD117^−^ cells were sorted from 2008 cells (Fig. 6e), and cultured for 12 days in the absence or presence of NCT-501 at 100 µM. Both the abundance of ALDH^+^ cells and the amount of de novo-produced CD44^+^CD117^+^ cells decreased after NCT-501 treatment (Fig. 6f and g). Given that CD44^+^CD117^+^ cells derived from the 2008 ovarian cancer cell line possess all CSC properties^18^, this result suggests that the ALDH1A1 inhibitor is also able to inhibit the ovarian cancer cell dedifferentiation. Taken together, our data clearly demonstrate that enhanced ALDH1A1 expression plays a crucial role in DDB2 silencing-promoted ovarian cancer cell dedifferentiation.

### ALDH1A1 inhibitor diminishes the CSC subpopulation in ovarian cancer cells possessing low DDB2 expression

**Fig. 7:**
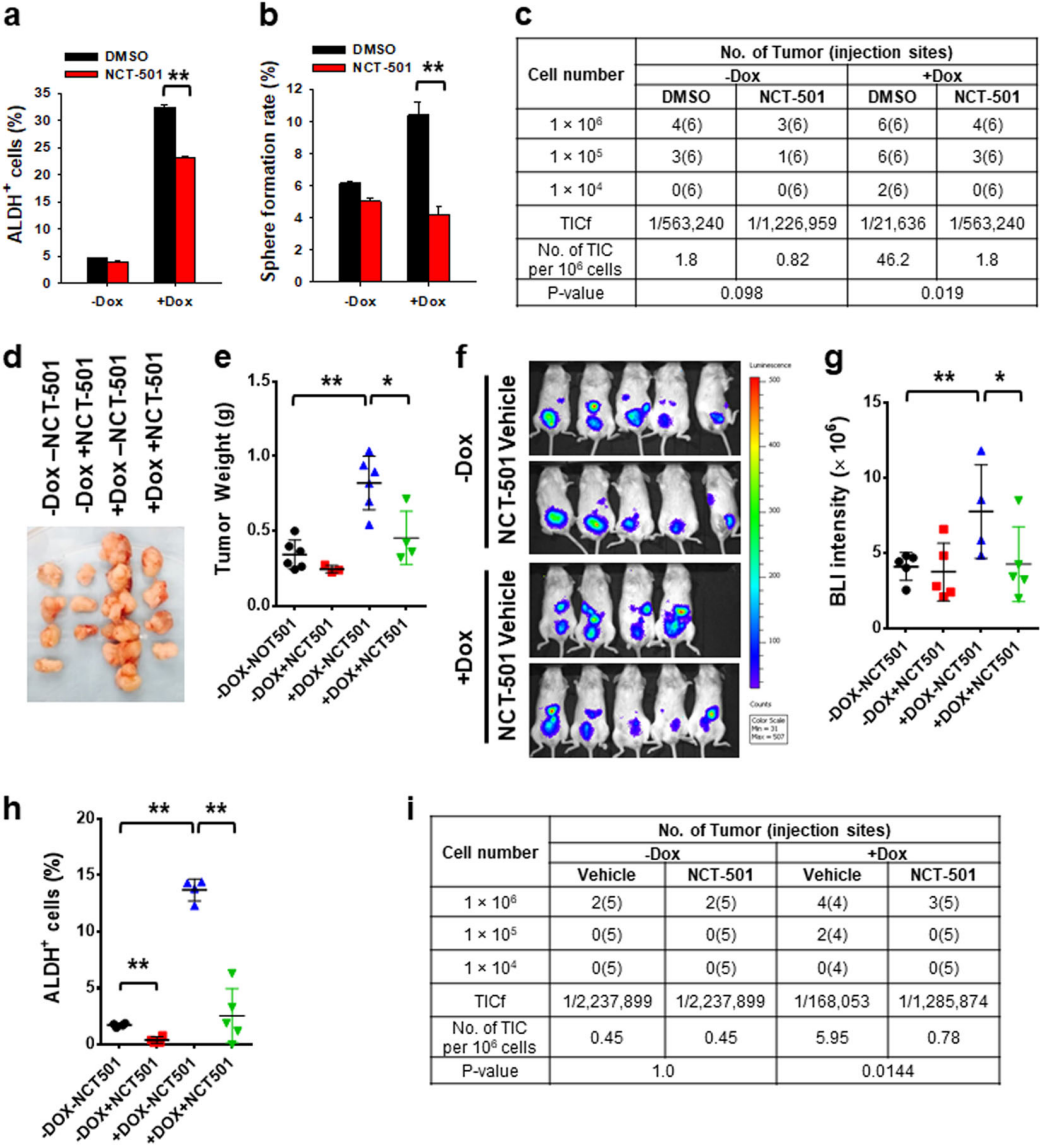
ALDH1A1 inhibitor antagonizes DDB2 silencing-induced CSC expansion and tumor growth. **a–e** 2008-pTRIPZ-shDDB2 cells were treated with Dox for 10 days and NCT-501 (100 nM) for 24 h in vitro. The percentage of ALDH^+^ cells (**a**) and their sphere formation rates (**b**) were determined using FACS and the sphere formation assay, respectively. *N* = 3, Bar: SD, **P < 0.01. The TICf was evaluated using the xenograft assay with limiting dilution (**c**). Tumor images (**d**) and weights (**e**) of xenografts generated with 1 × 10^6^ cells at the end of the xenograft experiment were shown. *P < 0.05, **P < 0.01. **f**–**i** Orthotopic ovarian xenografts were generated by intraperitoneally injecting 2008-pTRIPZ-shDDB2-Luc cells into NOD/SCID mice, and further treated with Dox or/and NCT-501 for 20 days. The volume of tumors was determined by BLI (**f**), and the BLI intensity was quantified (**g**). Xenograft tumors were isolated, the percentage of ALDH^+^ cells (**h**) and the TICf (**i**) in xenografts were determined. **P < 0.01

Given that the ALDH1A1 inhibitor NCT-501 can offset DDB2 silencing-induced non-CSC-to-CSC conversions, we reasoned that inhibition of ALDH1A1 activity is also able to reduce the CSC subpopulation and diminish their tumorigenicity in ovarian cancer cells carrying a low level of DDB2. We grew 2008-pTRIPZ-shDDB2 cells in the absence or presence of Dox, and treated cells with NCT-501 for 24 h. As expected, NCT-501 treatment reduced the DDB2 silencing-augmented ALDH^+^ cell subpopulation (Fig. 7a) and inhibited DDB2 silencing-promoted sphere formation capacity of these cells (Fig. 7b). We further examined the tumor-initiating potential of these cells using the subcutaneous xenograft assay. NCT-501 treatment of Dox-treated 2008-pTRIPZ-shDDB2 cells, which express downregulated DDB2, decreased their TICf by ~25-fold compared to DMSO treated cells (Fig. 7c). In addition, the volume of tumors derived from NCT-501 treated cells was smaller than that of DMSO treated cells in the presence of Dox (Fig. 7d, e). In contrast, NCT-501 treatment did not affect the percentage of ALDH^+^ cells, nor the sphere formation ability and tumorigenicity of non-Dox-treated cells (Fig. 7a–e). These results indicate that ALDH1A1 inhibition is only able to reduce the CSC subpopulation in ovarian cancers harboring low DDB2 expression.

To further confirm this finding, we generated orthotopic xenografts by injecting 2008-pTRIPZ-shDDB2-Luc cells into NOD/SCID mice intraperitoneally. These mice were treated with either Dox to induce DDB2 knockdown in xenograft tumor cells, or/and NCT-501 to inhibit the activity of ALDH1A1. Similar to the aforementioned in vitro study, in vivo treatment with Dox indeed increased the tumor size, while simultaneous treatment with Dox and NCT-501 reduced Dox-increased tumor growth (Fig. 7f, g). However, the NCT-501 treatment did not influence the tumor growth in non-Dox-treated mice (Fig. 7f, g). We further isolated xenograft tumor cells, confirmed the promoting effect of DDB2 silencing (+Dox) and inhibiting effect of NCT-501 on the abundance of ALDH^+^ cells using FACS (Fig. 7h), and analyzed the tumorigenic potential of these cells by determining the TICf. Again, we found that in vivo treatment of xenograft-bearing mice with Dox increased the TICf in the xenograft tumors, whereas concurrent treatment with Dox and NCT-501 offset Dox-induced increase of TICf (Fig. 7i). Taken together, these data indicate that inhibition of ALDH1A1 activity is able to reduce the CSC subpopulation, particularly in the ovarian cancer cell population with low DDB2 expression.

## Discussion

CSCs are believed to contribute to the tumor initiation, metastasis, and therapy resistance. Elimination of CSCs is considered an effective strategy to cure cancer, and this requires us to understand how the CSC subpopulation is maintained in tumors. Similar to normal stem cells, CSCs are also characterized by two key features, the capacity of self-renewal and differentiation. The balance between self-renewal and differentiation controls the abundance of CSCs in tumors. In addition, the non-CSCs can dedifferentiate and convert to CSCs under certain conditions to sustain the CSC pool^30^. We provide evidence in this study showing that this process also exists in ovarian cancer cells, and is controlled by the expression level of DDB2, e.g., high levels of DDB2 in ovarian cancer cells inhibit their capacity of dedifferentiation, halting the replenishment of CSCs, and hindering the ovarian cancer progression. Thus, the favorable prognosis of cancer patients with high DDB2 expression^15,16,18^ can be attributed to DDB2-promoted reduction of the CSC pool.

The DNA repair-independent role of DDB2 in suppressing cancer progression has been widely attributed to its transcription regulatory function. DDB2 has been recognized as a transcriptional regulator by directly binding to the promoter region of its target genes, and regulating the local histone modifications around the promoter region to affect the promoter activity of these genes^13,15–17,28,34,40^. The data presented here demonstrate that DDB2 is also able to bind to the *ALDH1A1* gene promoter and facilitate the enrichment of both EZH2 and histone H3K27me3 along the promoter region. In addition, we identified additional mechanism for transcription repression of the *ALDH1A1* gene by DDB2. DDB2 reduces the association of the transcription factor C/EBPβ to the *ALDH1A1* promoter by directly competing with C/EBPβ for binding. As a consequence, the transcription of the *ALDH1A1* gene is repressed in ovarian cancer cells possessing high levels of DDB2.

High ALDH activity is commonly found in various CSCs. ALDH activity not only represents a CSC marker, but also plays a critical role in maintenance of the CSC properties^23^. The data presented here further demonstrate that inhibition of ALDH activity through either downregulation of ALDH1A1 expression, or treatment with the ALDH1A1 selective inhibitor, blocked both sphere formation capacity and tumorigenic potential of ovarian cancer cells, supporting the function of ALDH activity in CSC subpopulation maintenance in tumors. However, the precise underlying mechanism is still unclear. As a member of the human ALDH superfamily, the main function of ALDH1A1 is to catalyze the oxidation of both endogenously and exogenously produced aldehydes to their respective carboxylic acids^41^. This ALDH-mediated detoxification of toxic aldehydes is believed to be responsible for drug resistance in ALDH^high^ gastric cancer cells^42^, but is far-fetched in explaining its role in maintaining the CSC subpopulation. ALDH1A1 is also involved in the metabolism of vitamin A by catalyzing the conversion of retinaldehyde to retinoic acid (RA), which is able to promote cell differentiation^43^, and is important in determining the fate of hematopoietic stem cells (HSCs)^44^. However, the function of ALDH in HSCs to promote differentiation via the production of RA contradicts its role in maintenance of the CSC properties, in particular its role in promoting the dedifferentiation of ovarian cancer cells demonstrated in the present study. Thus, we believe additional functions of ALDH1A1 are still undiscovered and warrant further investigation.

In summary, the data presented here demonstrate that DDB2 silencing is able to expand the CSC subpopulation by promoting cancer cell dedifferentiation. Enhanced ALDH1A1 expression due to DDB2 silencing-induced transcription de-repression plays a critical role in this process, and ALDH1A1 inhibition can block the non-CSC-to-CSC conversion, limit the CSC subpopulation, and halt the tumor regrowth in DDB2-downregulated ovarian cancer cells. These data provide a preclinical proof of concept for the ALDH1A1 inhibitor NCT-501 as a modality to improve the outcome of patients with ovarian cancers possessing low levels of DDB2.

## Materials and methods

### Cell culture and reagents

Human ovarian cancer cell line 2008^45,46^ was provided by Dr. Francois X. Claret (MD Anderson Cancer Center). SKOV3 and PEO4 cell lines were provided by Dr. Thomas C. Hamilton (Fox Chase Cancer Center). A2780/CP70 cell line was provided by Dr. Paul Modrich (Duke University). OVCAR3 cells were purchased from ATCC (Manassas, VA). CP70 cells stably transfected with pcDNA3.1-His-DDB2 (CP70-DDB2-1B) were established as described previously^12^. All cell lines were authenticated by STR profiling and tested for mycoplasma contamination. These cells were cultured in RPMI 1640 medium supplemented with 10% fetal bovine serum, 100 µg/mL streptomycin, and 100 units per mL penicillin. Doxycycline (Dox) was purchased from Sigma-Aldrich. Isopropyl β-d-1-thiogalactopyranoside (IPTG) was purchased from ThermoFisher. The ALDH1A1 selective inhibitor NCT-501 was provided by Dr. David Maloney. For treatment of in vitro-cultured cells, NCT-501 was dissolved in DMSO. For treatment of mice by intraperitoneal injection, NCT-501 was dissolved in 20% 2-hydroxypropryl-β-cyclodetrin (HPβCD) in saline.

### Plasmids, small-interfering RNA, cell transfection, and establishment of Tet- or IPTG-inducible stable cell lines

pReceiver-Lv105-DDB2 plasmids were constructed by GeneCopoeia. pcDNA3.1-His-DDB2 plasmids were generated in our laboratory^47^. siRNA SMARTpools designed to target human DDB2, a scramble non-targeting control small-interfering RNA (siRNA) (5′-UUCUCCGAACGUGUCACGU-3′), and Tet-On pTRIPZ-inducible shDDB2 plasmids (pTRIPZ-shDDB2) were purchased from Dharmacon. MISSION shDDB2 (TRCN0000083993), MISSION shALDH1A1 (TRCN0000026498), and IPTG-inducible pLKO-puro-IPTG-3 × LacOTR-shALDH1A1 constructs (TRCN0000026415) were purchased from Sigma. Full length of human DDB2 was cloned into a pTRE3G vector using In-Fusion HD Cloning System (Clontech) to generate Tet-On inducible pTRE3G-DDB2 constructs. All siRNA and plasmids were transfected into cells using Lipofectamine 2000 transfection reagent (Life Technologies).

To generate Tet-inducible or IPTG-inducible gene downregulation cell lines, 2008 cells were transfected with either pTRIPZ-shDDB2 or pLKO-puro-IPTG-3 × LacOTR-shALDH1A1 constructs, selected in the medium containing 2 µg/mL puromycin. The stably transfected cell lines (2008-pTRIPZ-shDDB2 and 2008-IPTG-shALDH1A1) were confirmed by western blotting. To generate the Tet-inducible DDB2-expressing cell line, SKOV3 cells were first transfected with pCMV-Tet3G plasmid (Clontech) and selected for a stable transfection clone with G418. This cell line was further transfected with pTRE3G-DDB2 plasmids, and the stably transfected clone (SKOV3-pTRE3G-DDB2) was selected and maintained in medium containing 2 µg/mL puromycin.

### RNA extraction and quantitative reverse-transcription PCR

Total RNA was extracted using Trizol reagent (Life Technologies). The first strand complementary DNA (cDNA) was generated by the reverse-transcription system (Promega) in a 20 µL reaction containing 1 µg of total RNA. A 0.5 µL aliquot of cDNA was amplified by Fast SYBR Green PCR Master Mix (Applied Biosystems) in each 20 µL reaction. PCR reactions were run on the ABI 7900 Fast real-time PCR system (Applied Biosystems) in the OSUCCC Nucleic Acid Core Facility. The primers used for the real-time RT-PCR are listed in Table S1.

### Immunoblotting

Whole-cell lysates were prepared by boiling cell pellets for 10 min in SDS lysis buffer (2% SDS, 10% glycerol, 62 mmol/L Tris·HCl, pH 6.8, and a complete miniprotease inhibitor mixture (Roche Applied Science)). After protein quantification, equal amounts of proteins were loaded, separated on a polyacrylamide gel, and transferred to a nitrocellulose membrane. Protein bands were immune-detected with appropriate antibodies (Table S2).

### Flow cytometry analysis and cell sorting

Anti-CD44-FITC, anti-CD117-PE and their corresponding isotype controls (BD Pharmingen) were used for flow cytometric analysis and cell sorting. Briefly, cells were incubated with antibodies on ice for 40 min in the dark. After washing with cold PBS, cells were resuspended in 200 μL PBS and subjected to FACS analyses on a BD FACS Aria III Flow Cytometer. The ALDEFLUOR Kit (Stemcell Technologies) was used for ALDH^+^ cell analyses and sorting. For each sample, one half of cells was treated with 50 mM diethylaminobenzaldehyde (DEAB) to define negative gates.

### Sphere-forming assay

A total of 10,000 cells were mixed with semisolid media (MethoCult H4100, Stemcell Technologies) containing serum-free DMEM/F12 medium supplemented with 20% knockout serum replacement, 20 ng/mL EGF, and 10 ng/mL bFGF (Life Technologies), and seeded in ultra-low attachment plates (Corning). Sphere formation was assessed 2 weeks after cell seeding.

### Luciferase reporter assay

CP70 and CP70-DDB2-1B cells were transiently transfected with pcDNA3.1-DDB2 plasmid and DDB2 siRNA, respectively, together with GoClone plasmids (SwitchGear Genomics) encoding *Renilla* luciferase with the *ALDH1A1* promoter region (−1~−1068). Cells were plated in 96-well plates after 24 h. Luciferase activity was detected 48 h after transfection using LightSwitch luciferase assay system (Active Motif) in plate luminometer (Promega). Relative luciferase units were calculated by subtracting background signal and normalizing *Renilla* signal to loading controls.

### ChIP assay

The ChIP assay was carried out using the ChIP-IT Express Enzymatic Kit (Active Motif) as described previously^13^. IP was performed with various ChIP-grade antibodies (Table S2). For IP of FLAG-tagged DDB2 from CP70 cells, EZview™ Red anti-flag-M2 affinity gel (Sigma-Aldrich) was used. Immunoprecipitated DNA was purified by phenol/chloroform extraction and quantified by ChIP-quantitative PCR (qPCR) analysis with primer sets corresponding to specific regions of the *ALDH1A1* gene promoter (Table S1). For the quantification of ChIP-qPCR, relative enrichment was calculated by normalization to input. In addition, fold enrichment was calculated by normalization to input first, then normalized to cells transfected with empty vector, which is set at 1.

### Electrophoretic mobility shift assay

IRDye 700 5′-end-labeled oligos (23–30 bp) flanking the putative DDB2 binding sites around P1, P2, and P7 regions in the *ALDH1A1* promoter were synthesized (Integrated DNA Technologies, Coralville, IA), annealed, and used as DNA probes. The annealing and binding assays were conducted according to the manufacturer’s instruction (Li-Cor, Lincoln, NE). Nuclear extracts were prepared from HeLa cells by lysing cells in the lysis buffer (50 mM Tris-HCl, pH7.4, 150 mM NaCl, 1 mM EDTA, 1% Triton X-100, and a protease inhibitor cocktail). EMSA assays were conducted in a 20 µL reaction, and the products were resolved in native 4% polyacrylamide gels at 10 V/cm at 4 °C in the dark in 0.5× Tris borate/EDTA buffer and imaged by Li-Cor Odyssey Imager (Li-Cor).

### Xenograft tumor study

NOD/SCID mice (6–8 week, female, 20–25 g body weight) were obtained from National Cancer Institute. Animals’ care was in accordance with institutional guidelines, and all studies were performed with approval of the Institutional Animal Care and Use Committee (IACUC) at the Ohio State University. To determine the frequency of tumor-initiating cells (TICf) using the limiting dilution assay (LDA), three cell doses (1 × 10^6^, 1 × 10^5^, 1 × 10^4^) of each sample were injected subcutaneously into the axillas of NOD/SCID mice. Mice were monitored for up to 4 weeks post injection, and the tumor number per group within this period was used to calculate the TICf using ELDA software (http://bioinf.wehi.edu.au/software/elda/index.html)^48^. After killing the mice, tumors were collected for subsequent studies or passaging.

For orthotopic xenograft and ALDH1A1 inhibitor treatment, GFP-tagged luciferase plasmids were transfected into 2008-pTRIPZ-shDDB2 ovarian cancer cells, and the stably transfected 2008-pTRIPZ-shDDB2-Luc cells were selected using FACS. A total of 5 × 10^6^ cells suspended in 100 µL PBS were injected into NOD/SCID mice intraperitoneally. Mice were divided into four groups after 1 week, and administrated with Dox (25 mg/kg, twice a week) or/and NCT-501 (10 mg/kg, every other day) intraperitoneally for 20 days. Mice in the control group were injected with vehicle reagents (20% HPβCD in saline). Bioluminescence imaging was carried out to show the xenografts. Mice were then killed, and xenograft tumor cells were isolated. The ALDH^+^ cells were determined using FACS, and the TICf in these xenografts were further determined using the subcutaneous xenograft assay with limiting dilution.

### Statistical analysis

Descriptive statistics, i.e., means ± SD, are shown on the figures. Two sample *t-*tests or analysis of variance were performed for data analysis for experiments with two groups or more than two groups’ comparisons. Generalized linear model as described by Hu and Smyth^48^ was used for the TICf analysis. For all statistical methods, *P* < 0.05 was considered statistically significant. All tests were two-sided. The animals were assigned randomly to the various experimental groups. Power analysis was used to calculate the sample size to provide at least 80% power to detect the specified differences. The studies reported here were not blinded. All experiments were run in triplicates, except specified otherwise.

## Electronic supplementary material

Supplementary Tables and Figures
