# Supplementary material for: DDB2 represses ovarian cancer cell dedifferentiation by suppressing ALDH1A1
Source: Cell Death Dis. 2018 May 11;9(5):561. doi: 10.1038/s41419-018-0585-y (PMC5948213; doi:10.1038/s41419-018-0585-y)
Supplement: Supplementary file 1 — Supplementary Tables and Figures [file 41419_2018_585_MOESM1_ESM.docx]

**Supplementary Tables**

**Table S1. Sequences of Primers and probes used in this study**

| **Primers/probes** | **Forward** | **Reverse** |
| --- | --- | --- |
| qRT-PCR Primers | | |
| ALDH1A1 | 5’- GCTTCCTGCCCTAGGTGTTAC-3’ | 5’- GCCACTCACTGAATGCC-3’ |
| DDB2 | 5’- CTCCTCAATGGAGGGAACAA-3’ | 5’- GTGACCACCATTCGGCTACT-3’ |
| GAPDH | 5’- AAGGTGAAGGTCGGAGT-3’ | 5’- GAAGATGGTGATGGGATTTC-3’ |
| ChIP-PCR Primers | | |
| P1 | 5’-GCTTCCTGCCCTAGGTGTTA-3’ | 5’-GAACACAGGTGACTGGCTCA-3’ |
| P2 | 5’-TGGAGCACTGGTTTCTTAAGG-3’ | 5’-GATTGGATGAACAAACTCAGAGC-3’ |
| P3 | 5’-TCTTCCCCAAACAGCACCTTG-3’ | 5’-AGGGCACTTGTTCTCAGTAGA-3’ |
| P4 | 5’-CCTGTCTCCAAAGCAACTCC-3’ | 5’-TCCAGGCGATGAAAGCTCAAG-3’ |
| P5 | 5’-GCCACCATGTGAAGAAGGAC-3’ | 5’-GGAGCCTTTGGGAGGTGTAT-3’ |
| P6 | 5’-GAGTGGACAGGTCAGGCTCT-3’ | 5’-TTGAAGCTATTGAAGGGGACCA-3’ |
| P7 | 5’-GCCAGCAGCCTGTACTCTGA-3’ | 5’-GCTCAGCATCATTCTGGTGTGAG-3’ |
| EMSA Probes | | |
| BS1 | 5’-IRDye700-AAATTGCTGAGCCAGTCACCTGTGTTCCAG-3’ | 5’-IRDye700-CTGGAACACAGGTGACTGGCTCAGCAATTT-3’ |
| BS7 | 5’-IRDye700-GCCAGCAGCCTGTACTCTGAGCC-3’ | 5’-IRDye700-GGCTCAGAGTACAGGCTGCTGGC-3’ |

**Table S2. Antibodies used in this study**

| **Antibody** | **Catalog number** | **Company** |
| --- | --- | --- |
| Western blotting | | |
| Anti-DDB2 | AF3297 | R&D Systems |
| Anti-ALDH1A1 | Ab53492 | Abcam |
| Anti-C/EBPβ | Sc-7962 | Santa Cruz |
| Anti-Lamin B | Sc-6216 | Santa Cruz |
| Anti-GAPDH | Sc-47724 | Santa Cruz |
| ChIP | | |
| Anti-C/EBPβ | Sc-7962 | Santa Cruz |
| Anti-H3K27me3 | 39155 | Active Motif |
| Anti-H3K9me3 | 39161 | Active Motif |
| Anti-EZH2 | 5246 | Cell Signaling |
| EZview Red anti-FLAG M2 Affinity Gel | F2426 | Sigma-Aldrich |
| Normal Rabbit IgG | Sc-2027 | Santa Cruz |

**Supplementary Figures**

**Figure S1.** Doxycycline-induced DDB2 silencing expanded the CSC subpopulation characterized by ALDH^+^ in ovarian cancer cells. **(a)** Dox treatment depleted DDB2 expression in 2008-pTRIPZ-shDDB2-c1 cells. **(b and c)** Dox treatment increased the ALDH^+^ cell population in 2008-pTRIPZ-shDDB2-c1 cells. (**d**) Dox treatment depleted DDB2 expression in 2008-pTRIPZ-shDDB2-c2 cells. (**e** and **f**) Dox treatment increased the ALDH^+^ cell population in 2008-pTRIPZ-shDDB2-c2 cells. (**g** and **h**) Subcutaneous xenograft assay shows that Dox treatment increased tumorigenic potential of 2008-pTRIPZ-shDDB2-c1 cells. Kaplan-Meier tumor-free survival summary plot for mice subcutaneously injected with Dox-treated or non-treated 2008-pTRIPZ-shDDB2-c1 cells (**g**). Mice were euthanized and tumors were weighed after 22 days of injection (h). **: P < 0.01.

**Figure S2.** Isolation and characterization of 2008-ALDH^+^ cells. **(a)** Ovarian cancer cell line 2008 cells were stained with ALDEFLUOR. ALDH^-^ (green) and ALDH^+^ (purple) cells were sorted with FACS. **(b)** RNA was extracted from 2008-ALDH^-^ and 2008-ALDH^+^ cells, and various stem cell markers were determined using real-time RT-PCR. Bar: SD, n=3, **: *P*<0.01. **(c)** Sphere formation of 2008-ALDH^-^ and 2008-ALDH^+^ cells was assessed as described in Materials and Methods. Bar: SD, n=3, **: *P*<0.01. **(d)** Various amounts of cells were mixed with Matrigel (1:1) and injected into NOD/SCID mice subcutaneously. The formation of tumor was observed up to 4 weeks.

**Figure S3.** Doxycycline does not affect the spontaneous conversion of ALDH^-^ to ALDH^+^ cells. **(a)** Ovarian cancer cell line 2008 were stained with ALDEFLUOR, and ALDH^-^ cells were sorted with FACS (purple). **(b, c)** ALDH^-^ cells were cultured in the absence or presence of Dox for 12 days. The percentage of ALDH^+^ cells (green) was determined using FACS (c,d). N = 3, Bar: SD, **: P < 0.01.

**Figure S4.** DDB2 inhibits the ALDH^-^-to-ALDH^+^ cells conversion in CP70 cells. **(a)** DDB2 and ALDH1A1 expression in C6 and C19 clones isolated from CP70 cells were determined using immunoblotting. **(b-d)** Conversion of ALDH^-^ cells to ALDH^+^ cells in the C6 and C19 clones. ALDH^-^ cells (purple) were sorted from CP70-C6 (b) and CP70-C19 (c) cells using FACS. These ALDH^-^ cells were cultured for 12 days, the percentage of ALDH^+^ cells (blue) was determined using FACS. ALDH^-^ cells isolated from the CP70-C19 clone were transfected with siDDB2 once every three days during 12 days’ culture, the percentage of ALDH^+^ cells was determined using FACS (d). **(e)** Quantitative assessment of de novo generated ALDH^+^ cells in C6 and C19 cell clones as well as the C19 clone transfected with DDB2 siRNA. N = 3, Bar: SD, *: P < 0.05, **: P < 0.01.

**Figure S5.** Selection of NCT-501 doses for experiments. **(a)** 2008 cells were treated with NCT-501 at different doses for 7 days, cell viability was determined using the MTT assay. **(b)** 2008 cells were treated with NCT-501 at different doses for 24 hours, the ALDH activity was determined with the ALDEFLUOR kit using FACS. N = 3.
